# The association between lipid metabolism and coronary artery disease: A systematic Mendelian randomization study combined with transcriptome analysis

**DOI:** 10.1097/MD.0000000000047880

**Published:** 2026-03-27

**Authors:** Qi Bure, Wenjin Sun, Lujiao Wang, Xin Zhang, Lian Shuang

**Affiliations:** a Emergency Department, Affiliated Hospital of Inner Mongolia Medical University, Hohhot City, Inner Mongolia Autonomous Region, China; b Center of Geriatric Medicine, Affiliated Hospital of Inner Mongolia Medical University, Hohhot City, Inner Mongolia Autonomous Region, China.

**Keywords:** biomarker, coronary artery disease, lipid metabolism, machine learning, Mendelian randomization

## Abstract

Coronary artery disease (CAD), a chronic progressive inflammatory cardiovascular disorder and leading global cause of mortality, imposes a substantial worldwide economic burden. Identifying lipid metabolism-related genes linked to CAD is crucial for deepening our understanding of the disease’s pathogenesis and discovering novel therapeutic targets. A total of 700 differentially expressed genes associated with CAD were determined by comparing CAD patients and healthy controls in the GSE250283 dataset. A positive relationship between lipid exposure and CAD was revealed by implementing a 2-sample Mendelian randomization analysis using genome-wide association studies data on lipid metabolism exposures and CAD outcomes. Further Mendelian randomization analysis, employing expression quantitative trait loci data from the identified differentially expressed genes as exposures and intersecting results with the Kyoto Encyclopedia of Genes and Genomes lipid metabolism pathway, identified 19 key genes exhibiting both lipid regulatory characteristics and reliable causal associations with CAD. Finally, 5 biomarker genes (SCP2, TNFAIP8, HMGCR, AGPAT3, and MAPKAPK2) were selected from the key genes by implementing 4 machine learning algorithms, and the developed nomogram incorporating these biomarkers demonstrated superior predictive accuracy for CAD risk stratification. The identification of these 5 genes as causal lipid metabolism biomarkers of CAD offers novel insights with high clinical potential, providing valuable targets for the management and treatment of CAD.

## 1. Introduction

Coronary artery disease (CAD), a chronic progressive inflammatory cardiovascular disorder, arises from partial stenosis or occlusion of coronary arteries that compromises myocardial blood supply, oxygen delivery, and nutrient provision. This vascular impairment leads to cardiac dysfunction and/or organic lesions, manifesting as angina pectoris or myocardial infarction – currently one of the leading causes of mortality worldwide and presents a huge global economic burden.^[1,2]^ According to the data from the Centers for Disease Control and Prevention (CDC) of the United States, cardiovascular diseases claimed 7,02,880 American lives in 2022, accounting for 20% of total deaths, with associated costs reaching 252.2 billion encompassing medical services, medications, and productivity losses.^[3]^ Research predictions show that the mortality rate of cardiovascular diseases in China will rise from 0.39% to 0.46% in 2021–2024, and then stabilize (0.44%) in 2030, indicating that the burden of CAD-related deaths in China will remain at a relatively high level in the coming years.^[4]^ The latest forecast shows that with the aging of the population and the increase of risk factors such as hypertension and obesity, by 2050, exceeding 61% of the people (more than 184 million) in the United States will suffer from cardiovascular disease, and the related costs are expected to be as high as 1.8 trillion US dollars.^[5]^ This exponential growth in healthcare expenditure underscores the critical urgency for enhanced CAD prevention and management strategies.

The pathogenesis of CAD is intrinsically linked to atherosclerosis – a core pathological process characterized by lipid deposition, inflammatory responses, fibrous tissue proliferation, and calcification.^[6]^ Dyslipidemia emerges as a pivotal driver, with chronic CAD patients frequently exhibiting adipose tissue degeneration and elevated lipid levels.^[7]^ Abnormal lipid profiles, particularly hypercholesterolemia, hypertriglyceridemia, and elevated low-density lipoprotein cholesterol (LDL-C), accelerate atherogenesis and CAD progression.^[8]^ The type of fatty acid also influences CAD risk. Zhuang et al demonstrated increased monounsaturated fatty acids elevate CAD risk whereas n − 3 polyunsaturated fatty acids confer protection.^[9]^ In addition, recent investigation found that lipid metabolic disturbances may alter gut microbiota composition, promoting proliferation of pathogenic bacteria that generate proinflammatory metabolites, thereby exacerbating atherosclerotic progression.^[10]^ These findings emphasize the therapeutic imperative of lipid profile regulation in CAD management and highlight the need for deeper mechanistic understanding of lipid metabolism-CAD interactions to identify novel therapeutic targets.

Genetic variations impact the occurrence and development of CAD through multiple pathways, among which lipid metabolism modulation is included. Existing researches revealed variants in PCSK9, LDL-R, APOB, APOE, and SORT1 correlate with LDL-C levels.^[11-14]^ For example, the polymorphism of the E670G locus of the PCSK9 gene exacerbating atherosclerosis severity via enhanced hepatic low-density lipoprotein (LDL) receptor degradation and impaired plasma LDL clearance.^[15]^ While genes such as LPL, APOA5, ANGPTL4, APOC3, and TRIB1 are related to triglyceride levels.^[11,13,14]^ For instance, Jørgensen et al conducted a prospective study in general Danish population and discovered 3 loss-of-function mutants in APOC3 (R19X, IVS2 + 1G→A, A43T) associated with 46% reduced plasma ApoC3 levels, 39% to 44% lower triglycerides, and 36% to 40% decreased CAD risk in heterozygotes.^[16]^ These genetic variations not only deepen our understanding of the pathogenesis of CAD, but also can be used as tools for the early diagnosis and risk prediction of CAD. However, despite the existence of genetic predictors, the rising prevalence of CAD requires the discovery of novel biomarkers for early diagnosis, risk stratification, and therapeutic innovation.

Expression quantitative trait loci (eQTLs) represent genetic variants statistically associated with gene expression levels, serving as critical tools for deciphing genotype-phenotype correlations.^[17]^ In causal inference methodologies, genetic variants have been effectively employed as instrumental variables (IVs) to emulate the biological effects of modifiable risk factors on disease susceptibility through Mendelian randomization (MR).^[18]^ This approach offers unique advantages in observational research by leveraging genetic predisposition to minimize residual confounding and reverse causation biases, thereby enabling robust causal inference between exposures and clinical outcomes.^[19,20]^ Our integrative analytical framework synergizes transcriptomic profiling with MR methodology to systematically investigate the causal contributions of lipid metabolism biomarkers to CAD pathogenesis. The experimental pipeline comprised 3 principal phases: first, differentially expressed genes (DEGs) associated with CAD were identified through comprehensive transcriptome analysis. Second, a 2-sample MR analysis were implemented using lipid metabolism exposure data and CAD outcome statistics derived from genome-wide association studies (GWAS) to establish the causal relationship between lipids and CAD. Finally, we conducted a secondary MR analysis utilizing eQTL data from identified DEGs as exposures, followed by pathway enrichment analysis through intersection with the Kyoto Encyclopedia of Genes and Genomes (KEGG) lipid metabolism gene set, thereby pinpointing dual-function genes demonstrating both lipid regulatory properties and CAD associations.

Machine learning (ML), a transformative artificial intelligence subset, employs sophisticated mathematical models to extract complex patterns from multidimensional data, enabling breakthroughs in predictive analytics, anomaly detection, and pattern recognition.^[21]^ Capitalizing on these capabilities, our study implemented ensemble ML algorithms to optimize biomarker selection from candidate genes. The final predictive framework integrates these biomarkers through multivariate logistic regression into a clinical diagnostic nomogram, creating a precision medicine tool designed for CAD risk stratification and preventive intervention. This integrative computational framework not only advances personalized cardiovascular medicine but also holds translational potential for mitigating CAD burden through early detection and tailored therapeutic strategies.

## 2. Materials and methods

### 2.1. Study design

This study was approved by the Ethics Committee of Affiliated Hospital of Inner Mongolia Medical University. We performed a 2-sample MR analysis integrated with transcriptomic profiling to investigate causal relationships between lipid metabolism-associated gene expression and CAD. The current study consists of 4 sequential phases – Transcriptomic profiling: CAD-associated DEGs were identified through comparative analysis of transcriptomic data from CAD patients and healthy controls in the Gene Expression Omnibus (GEO) database; causal MR analysis: we applied MR analysis to investigate the causal effects of lipid metabolism on CAD using publicly available GWAS datasets; transcriptome-MR integration: a synergistic approach combining transcriptomic and MR analyses was employed to identify key CAD-influencing genes (the genes are both DEGs and involved in the lipid metabolism pathway); predictive model construction: 4 ML algorithms (Least Absolute Shrinkage and Selection Operator [LASSO] Regression, Random Forest, Support Vector Machine (SVM), and Extreme Gradient Boosting [XGBoost]) were used to evaluate the identified key genes, and consensus analysis of model outputs determined 5 high-confidence biomarkers as robust CAD predictors for the final logistic clinical nomogram model development.

### 2.2. Transcriptomic analysis

Transcriptome sequencing data were obtained from the GEO public database (https://www.ncbi.nlm.nih.gov/geo). There were 56 peripheral blood samples from the GSE250283 dataset,^[22]^ comprising 41 CAD patients and 15 healthy controls, with a cohort composition of 36 males and 20 females. The raw data underwent the following preprocessing pipeline: initially, quality control filtering was performed to remove outliers and missing values, followed by data standardization using quantile normalization method. Based on microarray probe annotation information, probe IDs were converted to standard gene symbols, and protein-coding genes were selected for subsequent bioinformatics analysis.

Differential expression analysis between CAD patients and controls was conducted through Limma (version: 3.50.1) in the R package (Vienna, Austria), with statistically DEGs identified using stringent thresholds: absolute log_2_ fold change > 0.2 (|log_2_ FC| > 0.2) and adjusted *P* < .05. Finally, volcano plots visualizing DEGs were generated using the ggplot2^[23]^ in R package to illustrate gene expression differences between groups.

To systematically investigate the biological significance of the DEGs, we conducted Gene Ontology (GO) and KEGG pathway enrichment analyses using the clusterProfiler package (version: 4.2.2)^[24]^ in R. These analyses were performed with a statistically significant threshold set at *P* < .05 to identify biologically relevant functional categories and signaling pathways associated with the DEGs. The top 20 most important functions enriched in GO and KEGG were visualized using the R pakage enrichment plot (version: 1.18.0). A gene set enrichment analysis was performed based on the |log_2_ FC| sequences of all the DEGs using the gene set of “lipid metabolism” from the MSigDB database (https://www.gsea-msigdb.org/gsea/msigdb/) as reference gene set. The screening criteria were adjusted to *P* < .05.

### 2.3. MR analysis of lipid exposure and CAD

We implemented a 2-sample MR framework to investigate putative causal relationships between lipid metabolism and CAD risk. The analysis utilized GWAS data from 2 independent consortia: exposure instruments were derived from the Global Lipids Genetics Consortium (GLGC 2013, accession ieu-a-300), comprising 1,73,082 samples with 24,37,752 genotyped single nucleotide polymorphisms (SNPs); outcome data were obtained from the Coronary Artery Disease Genome-wide Replication and Meta-analysis consortium (CARDIoGRAM 2015, accession ieu-a-7), encompassing 1,84,305 samples with 94,55,779 imputed SNPs.

The MR design estimated causal effects of an exposure on an outcome by employing SNPs as IVs. The screening criteria for IVs are: SNPs significantly associated with lipid metabolism with a genome-wide significance level of *P* < 5 × 10^−8^; independent SNPs were retained for subsequent analysis with stringent parameters (*r*^2^ < 0.001 within a 10,000 base-pair windows) in linkage disequilibrium clumping.

Causal estimates were generated through 5 algorithms – inverse variance weighted (IVW),^[25]^ MR-Egger,^[26]^ weighted median,^[27]^ simple mode,^[28]^ and weighted mode.^[29]^ Sensitivity analyses (Cochran’s *Q* statistical test, MR-Egger test, and leave-one-out analysis) were performed to assess the robustness of the MR analysis results. The sensitivity analysis comprised the following components: heterogeneity testing was conducted using Cochran’s *Q* statistic (where *P* < .05 indicates significant heterogeneity); pleiotropy was assessed using the MR-Egger intercept test, where a statistically significant deviation of the intercept from zero (*P* < .05) indicates potential horizontal pleiotropy, thereby casting doubt on the validity of causal inference outcomes; and outlier detection was implemented using the leave-one-out analysis, with 10,000 permutations executed to validate the robustness of the results.

### 2.4. Transcriptomic-MR integrations

We performed an integrative causal inference analysis by synergizing MR frameworks with transcriptomic profiling to systematically identify effector genes influencing CAD pathogenesis. The analytical workflow proceeded as follows: First, tissue-specific eQTL data were acquired from the Genotype-Tissue Expression (GTEx) database (v8), filtering SNPs demonstrating genome-wide significant associations with DEGs’ expression at *P* < 5 × 10^−8^, alongside their genomic coordinates and functional annotations. Second, to ensure robust IV selection for MR, we computed critical genetic metrics – including effect size estimates (β), linkage disequilibrium-adjusted coefficients of determination (*R*^2^), standard errors, instrument strength indices (*F*-statistics > 10), and effect allele frequencies – for all DEG-linked eQTLs. Third, causal relationships between eQTL-regulated gene expression (exposure data) and CAD susceptibility (outcome data: ieu-a-7) were interrogated using IVW regression as the primary MR method, supplemented by sensitivity analyses (Cochran’s *Q* test, MR-Egger intercept test and leave-one-out analysis) to evaluate pleiotropy and outlier effects. Finally, the MR-derived CAD-associated genes were cross-referenced against 757 lipid metabolism pathway genes curated from the KEGG database, with statistically significant overlaps (*P* < .05) prioritized as functionally key candidates for downstream predictive modeling.

### 2.5. Machine learning-driven predictive modeling for CAD risk stratification

Our predictive modeling pipeline employed the key candidate genes exhibiting dual lipid metabolism-CAD associations as input features. Feature selection was conducted through 4 complementary ML algorithms: Random Forest (1000 decision trees, Gini index splitting, 10-fold cross-validation); XGBoost (learning rate = 0.1, max_depth = 10, early stopping at 50 iterations); SVM (Radial basis function kernel, cost = 1); and LASSO Regression (λ optimization via minimum MES [mean squared error] criterion in 10-fold cross-validation). The GSE250283 cohort (n = 56 samples) was partitioned into training (70%, n = 39) and validation (30%, n = 17) sets while preserving class proportions. Model discrimination was quantified by the area under the receiver operating characteristic curve (AUC-ROC, AUC > 0.7) with 95% confidence intervals (CI) derived from 1000 bootstrap resamples. Overlapping feature genes were acquired in all algorithms as predictors for the construction of subsequent logistic regression model.

A multivariable logistic regression model was built in the GSE250283 dataset based on the expression levels of these feature genes using the rms package (version 6.5-0) in R (v4.2.2). The model generated a clinical nomogram assigning weighted points based on standardized expression *z*-scores, translating total scores into CAD probabilities.

### 2.6. Statistical analysis

All analyses were performed in R v4.2.2. Multiple testing correction was applied using the Benjamini–Hochberg method^[30]^ to calculate the false discovery rate for adjusting *P* values. MR analyses utilized the TwoSampleMR and MR-PRESSO packages.

## 3. Results

### 3.1. Transcriptomic analysis of DEGs between CAD and control samples

**Figure 1.**
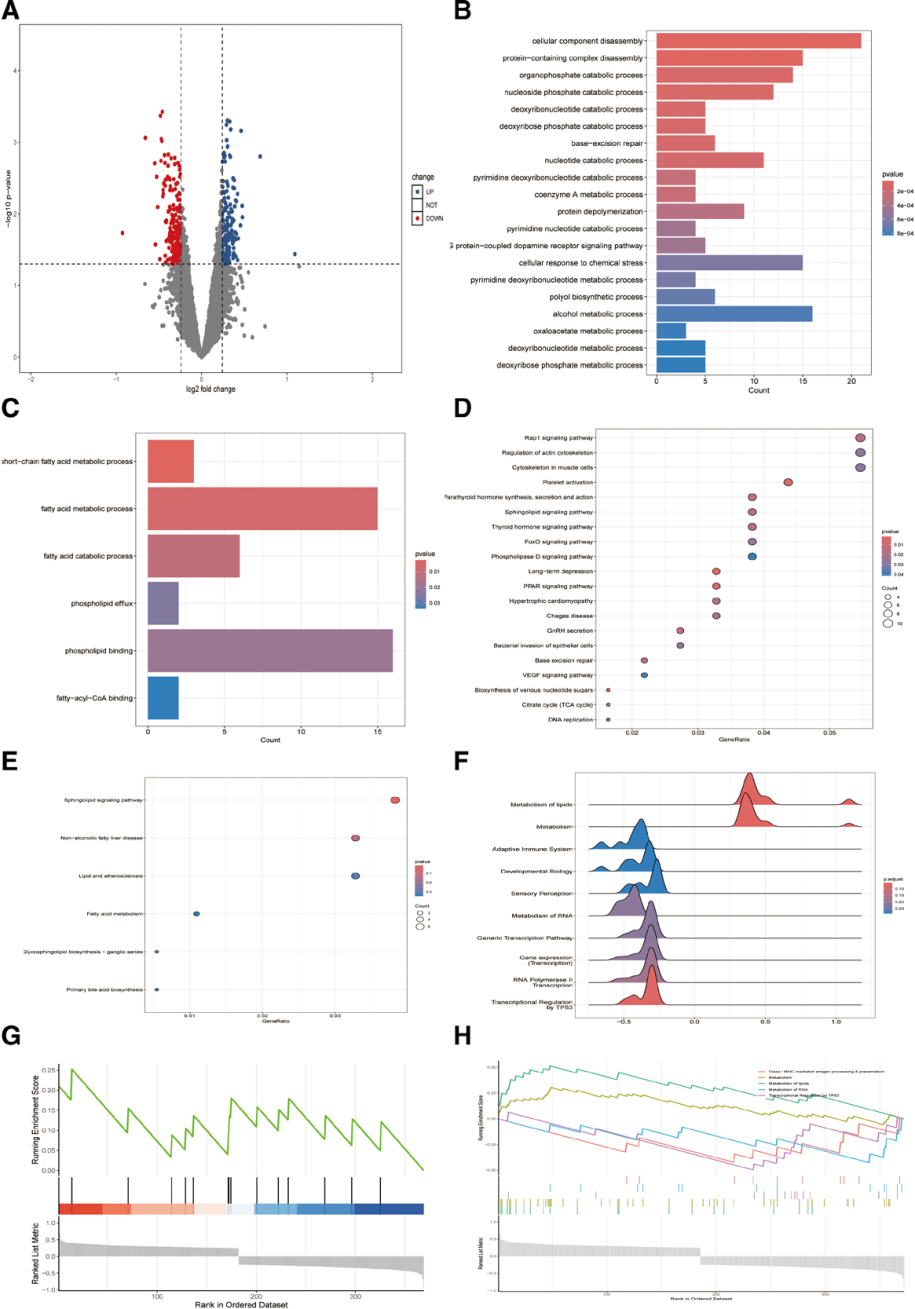
Transcriptomic analysis of differentially expressed genes in CAD and control samples. (A) Volcano plot depicting differentially expressed genes (DEGs) between CAD patients and controls. (B) Bar plot of the top 20 significantly enriched GO biological processes for DEGs. (C) Key selected lipid metabolism-related biological processes. (D) Dot plot of the top 20 enriched KEGG signaling pathways for DEGs. (E) Key selected lipid metabolism-associated signaling pathways.(F–H) Functional enrichment analysis of CAD-associated pathways by GSEA. CAD = coronary artery disease, DEG = differentially expressed gene, GO = Gene Ontology, GSEA = gene set enrichment analysis, KEGG = Kyoto Encyclopedia of Genes and Genomes.

By querying the GEO public database, this study obtained a transcriptomic sequencing dataset (Accession: GSE250283) comprising peripheral blood samples from 41 CAD patients and 15 healthy controls, totaling 56 samples for differential expression analysis. The findings demonstrated that: a total of 700 DEGs were identified between CAD patients and healthy controls, with 376 genes upregulated and 324 genes downregulated in CAD patients (Fig. 1A, Table S1, Supplemental Digital Content, https://links.lww.com/MD/R575); GO enrichment bar plot showed the top 20 significantly enriched biological processes (Fig. 1B), including cellular component disassembly, protein-containing complex disassembly, organophosphate catabolic process, nucleoside phosphate catabolic process, and deoxyribonucleoside catabolic process, etc. Notably, lipid metabolism-related processes such as short-chain fatty acid metabolic process, fatty acid metabolic process, and fatty acid catabolic process were prominently enriched (Fig. 1C); KEGG enrichment dot plot highlighted 20 key pathways (Fig. 1D), such as Rap1 signaling pathway, biosynthesis of various nucleotide sugars, platelet activation, peroxisome proliferator-activatedreceptorsignaling pathway, and parathyroid hormone synthesis/secretion/action, etc. Of particular interest, pathways related to lipid metabolism, such as sphingolipid signaling pathway, nonalcoholic fatty liver disease, and glycosphingolipid biosynthesis-ganglio series showed significant enrichment (Fig. 1E); Gene set enrichment analysis results (Figs. 1F-H) further confirmed substantial associations between CAD pathogenesis and lipid metabolic pathways, as well as cancer-related signaling pathways involving TP53-mediated transcriptional regulation.

### 3.2. MR analysis revealed a positive relationship between lipid exposure and CAD

**Figure 2.**
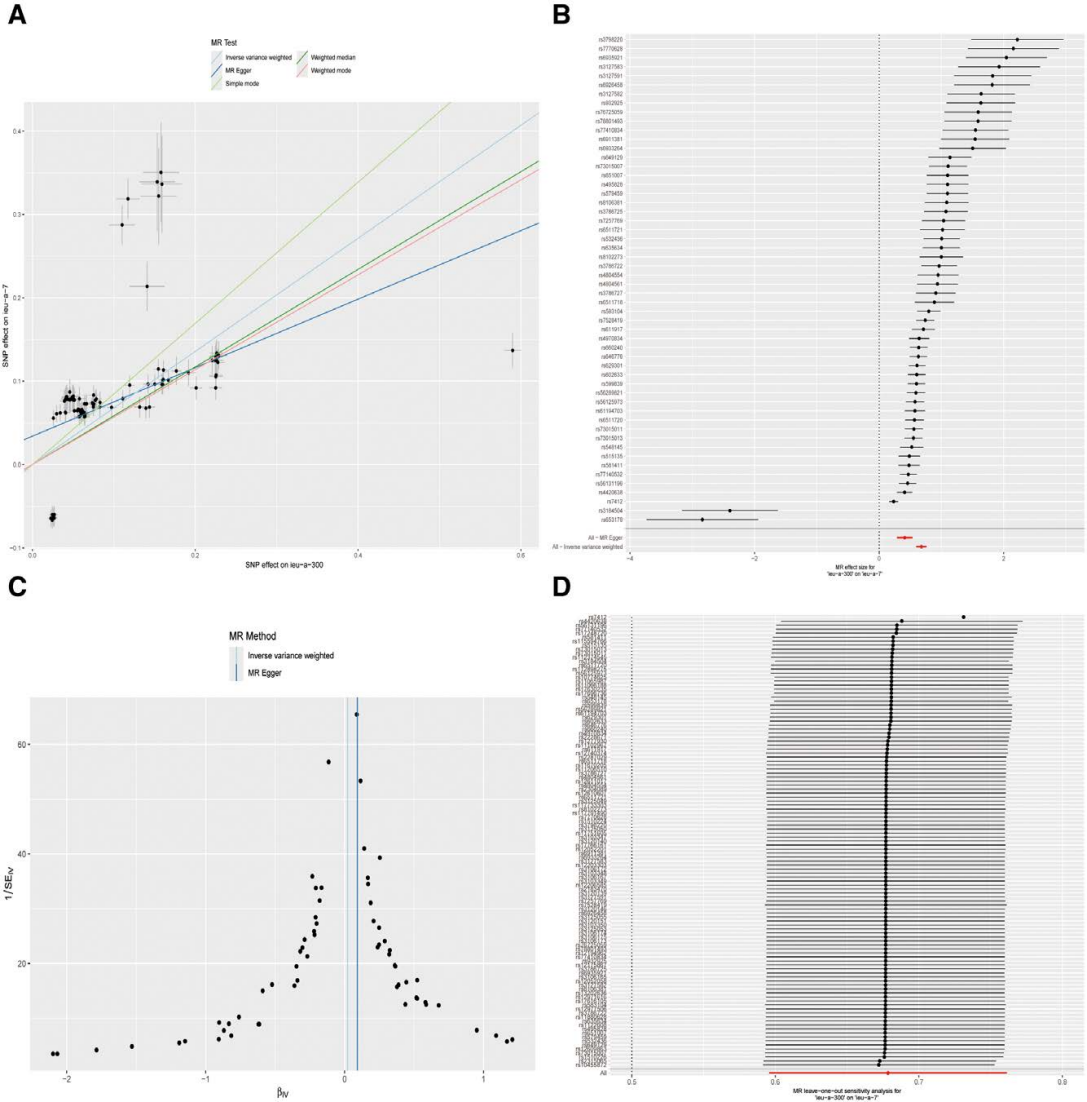
SNPs related to CAD risk. (A) Scatterplot of forward MR analyzing the effect of instrumental variables (SNPs) on exposure factors (lipid level) and the effect of instrumental variables (SNPs) on outcome (CAD). (B) Forest plot of effect sizes of exposure factors on outcome variables analyzed by MR using IVW method. (C) Funnel plot of instrumental variables for MR. (D) Leave-one-out analysis of instrumental variables for MR. CAD = coronary artery disease, IVW = inverse variance weighted, MR = Mendelian randomization, SNP = single nucleotide polymorphism.

This study identified 113 lipid-associated valid SNPs as IVs. MR analyses using IVW, MR-Egger, weighted median, simple mode, and weighted mode approaches consistently demonstrated significant causal relationships between lipid levels and CAD risk (all *P* < .05; Table 1). Scatter plots revealed concordant directional effects (β-values) across all 5 analytical methods, indicating a positive association between elevated lipid levels and CAD susceptibility (Table 1, Fig. 2A).

**Table 1:** Results of MR analysis between lipid levels and CAD risk.

Outcome	Exposure	Method	nSNP	β	SE	*P*
ieu-a-7	ieu-a-300	MR-Egger	113	0.410885076	0.063061352	2.20E−09
		WM	113	0.585383846	0.020517942	4.93E−179
		IVW	113	0.678496325	0.042268463	5.53E−58
		Simple mode	113	0.846504958	0.049777956	8.21E−33
		Weighted mode	113	0.569022529	0.019130049	5.46E−55

CAD = coronary artery disease, IVW = inverse variance weighted, MR = Mendelian randomization, nSNP = number of single nucleotide polymorphism, SE = standard error, WM = weighted median, β = effect size.

The forest plots based on IVW analysis showed that SNPs with MR effect estimates distributed to the left of 0 (negative effects) suggested a protective role of lipid elevation against CAD risk, while those to the right of 0 (positive effects) implied increased CAD risk. The aggregated effect (red diamond) ultimately demonstrated an overall positive correlation between lipid accumulation and CAD risk, confirming the aforementioned findings. Notably, no individual SNP exhibited disproportionately strong associations with CAD risk (Fig. 2B).

Funnel plot symmetry for IVW analysis indicated minimal bias in causal estimates (Fig. 2C). Leave-one-out sensitivity analysis revealed that no single SNP significantly influenced the causal estimates, with most IVs showing effect sizes closely aligned with the pooled estimates (Fig. 2D). These findings collectively confirm the robustness of the MR results, suggesting that the observed lipid-CAD causality is not driven by any single IV.

### 3.3. A total of 19 key genes of CAD were acquired by transcriptomic-MR integration

**Figure 3.**
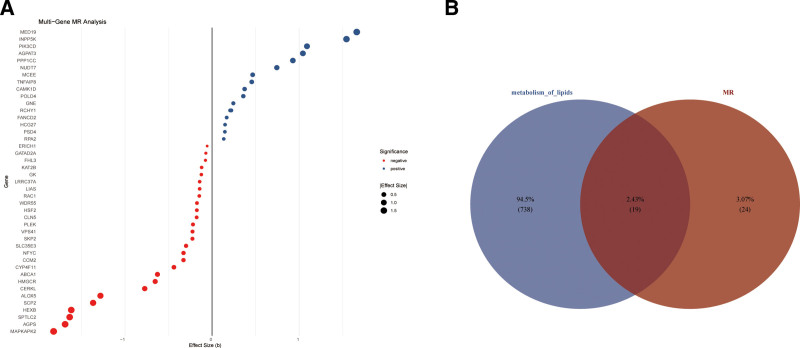
Key genes associated to CAD were acquired by transcriptomic-MR integration. (A) Effect sizes (β-values) of exposure factors (causal genes) on outcome (CAD) variables analyzed by MR using IVW method. (B) Venn plot shows the cross-results between causal genes (n = 44) and 757 lipid metabolism-related genes curated in the KEGG database. CAD = coronary artery disease, IVW = inverse variance weighted, KEGG = Kyoto Encyclopedia of Genes and Genomes, MR = Mendelian randomization.

Following rigorous screening procedures, 628 SNPs demonstrating strong associations with DEGs were identified as valid IVs. Utilizing a 2-sample MR approach with the IVW method as the primary analytical framework, we systematically evaluated the impact of each causal SNP locus on CAD susceptibility. This investigation revealed 44 CAD-associated genes, comprising 27 positively correlated and 16 negatively correlated candidates (Fig. 3A). To biologically contextualize these findings, we performed intersection analysis between these causal genes (n = 44) and 757 lipid metabolism-related genes curated in the KEGG database. This cross-validation yielded 19 consensus key genes demonstrating both statistical causality and pathway relevance (Fig. 3B), which were subsequently prioritized for ML-based predictive modeling. The causal relationship analysis results of these key genes and CAD were shown in Table 2.

**Table 2:** Results of MR analysis between 19 key genes and CAD risk.

Outcome	Exposure	Method	nSNP	β	SE	*P*
ieu-a-7	GK	IVW WM	2	−0.123	0.0439	.005
	ALOX5	IVW	3	−1.282	0.4450	.003
	AGPS	IVW	2	−1.688	0.4056	3.15e−05
	HMGCR	IVW MR_Egger WM	4	−0.653	0.1087	1.84e−09
	MAPKAPK2	IVW WM	2	−1.820	0.1950	1.02e−20
	TNFAIP8	IVW	2	0.4557	0.1658	.005
	HEXB	IVW MR_Egger	3	−1.617	0.3335	1.24e−06
	NFYC	IVW WM	2	−0.328	0.0309	3.05e−26
	CYP4F11	IVW WM	4	−0.438	0.1299	.0007
	SPTLC2	IVW	2	0.084	0.4401	.0044
	SCP2	IVW MR_Egger WM	4	−0.006	0.1254	.0005
	PPP1CC	IVW WM	2	0.9279	0.1045	7.13e−19
	AGPAT3	IVW MR Egger	2	1.043	0.0975	1.03e−26
	PIK3CD	IVW WM	3	1.090	0.1925	1.493e−08
	NUDT7	IVW	3	0.743	0.2037	.0002
	INPP5K	IVW WM	4	−0.029	0.1527	.0003
	MED19	IVW WM	5	−0.015	0.0983	.0004
	ABCA1	IVW MR_Egger	2	−0.118	1.5385	.0006
	MCEE	IVW WM	3	0.466	0.1791	.0042

CAD = coronary artery disease, IVW = inverse variance weighted, MR = Mendelian randomization , nSNP = number of single nucleotide polymorphism, SE = standard error, WM = weighted median, β = effect size.

### 3.4. A CAD risk prediction nomogram model was constructed based on 5 key biomarkers

**Figure 4.**
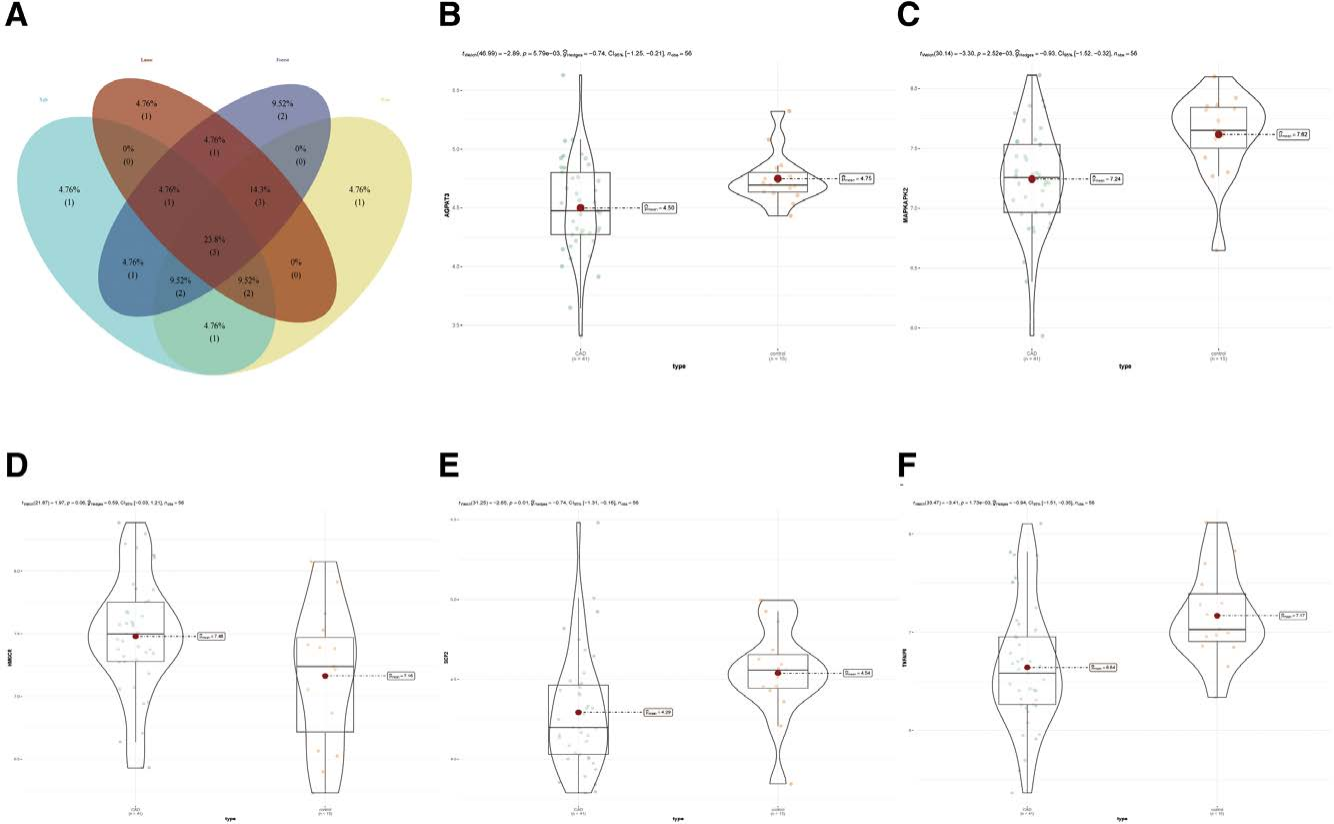
Five biomarkers were acquired and their expression patterns. (A) Five consensus biomarkers were acquired by 4 methods of machine learning. (B) The expression of AGPAT3 in CAD patients and control individuals. *P < .05; **, *P* < .01. (C) The expression of MAPKAPK2 in CAD patients and control individuals. *P < .05; **, *P* < .01. (D) The expression of HMGCR in CAD patients and control individuals. *P < .05; **, *P* < .01. (E) The expression of SCP2 in CAD patients and control individuals. *P < .05; **, *P* < .01. (F) The expression of TNFAIP8 in CAD patients and control individuals. *P < .05; **, *P* < .01. CAD = coronary artery disease.

To establish a more precise prognostic model, we employed 4 ML algorithms (Random Forest, XGBoost, SVM, and LASSO Regression) to further refine these critical genes. Intersection analysis identified 5 consensus biomarkers (Fig. 4A): SCP2, TNFAIP8, HMGCR, AGPAT3, and MAPKAPK2. Subsequent multivariate logistic regression analysis incorporating these biomarkers revealed SCP2 (odds ratio [OR] = 0.141, 95% CI: 1.345, *P* < .05), TNFAIP8 (OR = 0.024, 95% CI: 0.491, *P* < .05), HMGCR (OR = 0.589, 95% CI: 4.765, *P* < .05), and MAPKAPK2 (OR = 0.052, 95% CI: 0.898, *P* < .05) as independent risk factors for CAD (Table 3). Differential expression patterns of these 5 genes between CAD patients and controls are illustrated in Figure 4B–F. Among them, AGPAT3, MAPKAPK2, SCP2, TNFAIP8 exhibited significantly reduced expression levels, whereas HMGCR displayed elevated expression in CAD patients compared to healthy controls.

**Table 3:** Multivariable logistic regression analysis based on 5 key biomarkers.

	Coef	SE	Wald	*P*	OR	95% CI
SCP2	−1.9551	1.1489	-1.7	.0088	0.141	1.345
TNFAIP8	−3.7448	1.6397	-2.28	.0224	0.024	0.491
HMGCR	−0.5292	1.0702	-0.49	.0210	0.589	4.765
AGPAT3	−2.275	1.9423	-1.17	.2415	0.103	5.604
MAPKAPK2	−2.9496	1.3894	-2.12	.0338	0.052	0.898

CI = confidence interval, Coef = regression coefficient, OR = odds ratio, SE = standard error; Wald = Chi-square value.

**Figure 5.**
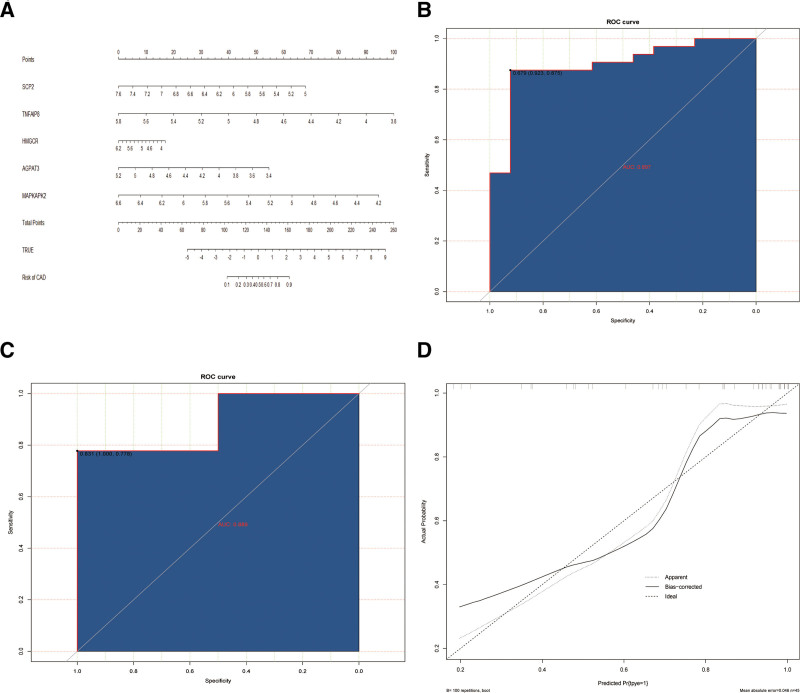
The construction and evaluation of nomogram model. (A) A diagnostic nomogram model of CAD risk prediction based on the expression of 5 biomarkers. (B) ROC plot for the GSE250283 training set. (C) ROC plot for the GSE250283 validation set. (D) Calibration decision curves for line diagram model. “Apparent” represents the actual curve, “Bias-corrected” represents the calibration curve, and “Ideal” represents the ideal curve. CAD = coronary artery disease, ROC = receiver operating characteristic.

A diagnostic nomogram integrating these biomarkers was constructed to predict CAD probability (Fig. 5A). Each risk factor (gene) was assigned a weighted score in the nomogram, with cumulative scores serving as a composite predictor of CAD probability for individual patients. The model demonstrated robust performance with AUC values of 0.897 and 0.889 in the GSE250283 training and validation sets, respectively (Fig. 5B, C). Calibration curves exhibited excellent concordance with the ideal reference line (Fig. 5D), indicating high predictive accuracy. These results collectively demonstrate the strong predictive capability of our CAD risk scoring model and highlight the critical role of these 5 biomarkers in CAD pathogenesis.

## 4. Discussion

As predominant contributors to global disease burden, cardiovascular diseases (CVD) exhibit CAD as their most prevalent clinical manifestation, exerting substantial socioeconomic pressures on healthcare systems worldwide.^[31]^ Early identification of CAD and its associated risk factors, along with effective interventions, is crucial for reducing its incidence and mortality rates. Dysregulated lipid homeostasis constitutes a pivotal etiological mechanism in CAD pathogenesis. Despite the existing therapeutic advances,^[32-34]^ escalating CAD incidence underscores the urgent need for elucidating metabolic dysregulation patterns and establishing robust predictive biomarkers. In the present study, we investigated the lipid metabolism-associated genes that contribute to CAD. By performing a integrative transcriptomic-MR analysis and implementing ML algorithms to optimize biomarker selection, 5 biomarker genes, SCP2, TNFAIP8, HMGCR, AGPAT3, and MAPKAPK2, were confirmed to be risk factors for CAD. The developed nomogram incorporating these biomarkers demonstrated superior predictive accuracy for CAD risk stratification, offering novel insights into precision prevention strategies. Although the predictive nomogram demonstrated promising diagnostic performance in the current dataset, further validation in larger, independent cohorts is necessary before clinical translation. Prospective studies integrating multi-center transcriptomic data, proteomics, and clinical outcomes would provide stronger evidence for the clinical applicability of these biomarkers.

Accumulating epidemiological evidence substantiates the causal link between dyslipidemia and CVD,^[35,36]^ a relationship corroborated by our MR analysis demonstrating a positive association between lipid exposure and CAD. The pro-atherogenic effect of elevated lipids predominantly stem from increased plasma LDL-C concentrations.^[36]^ Therefore, reducing plasma LDL-C has been the focus of cardiovascular risk mitigation strategies for decades. The cholesterol biosynthesis rate-limiting enzyme HMGCR (3-hydroxy-3-methylglutaryl-CoA reductase) has been reported to be overexpressed in various cancers (e.g., prostate, gastric, and colon carcinomas), and implicated in tumor progression.^[37-40]^ Notably, our study reveals that HMGCR also exhibit a significant upregulation in CAD patients and serves as an independent risk factor for CAD prediction. Statins, as targeted inhibitors of HMGCR, are first-line medications for lowering circulating LDL-C levels, and they can reduce the risk of CVD substantially.^[41]^ Therefore, since the advent of statins, the outcomes of CVD have significantly improved. Recent epidemiological data revealed that over 25% of US adults aged 40 years or older use statin therapy for CVD prevention.^[42]^

MAPKAPK2, a member of the mitogen-activated protein kinase (MAPK) superfamily, is a serine/threonine kinase widely present in mammalian cells.^[43]^ As a key effector molecule of the p38 MAPK signaling pathway, it participates in various biological functions, including regulating inflammatory responses, cell migration/proliferation, and modulating lipid and glucose metabolism.^[44,45]^ Numerous studies have demonstrated that upon phosphorylation and activation by p38 MAPK, MAPKAPK2 mediates the biosynthesis and signaling of inflammatory cytokines (TNF-α and IL-6).^[46,47]^ Notably, IL-6, a central mediator in inflammatory cascade, plays pivotal roles in the initiation and progression of atherosclerosis.^[48]^ Altered MAPKAPK2 expression signifies disruption of the metabolic-immune bistable equilibrium. Scharf et al discovered that mice lacking both MAPKAPK2 and MAPKAPK3 displayed differences in the expression of various genes involved in lipid and carbohydrate metabolism in skeletal muscle.^[49]^ Ozcan et al demonstrated that MAPKAPK2 inhibitors improved metabolism and enhanced atherosclerotic plaque stability in subjects with type 2 diabetes.^[50]^ To date, MAPKAPK2 has been identified as a biomarker or predictor in multiple pathologies including aortic dissection (AD), metabolic syndrome, and septic cardiomyopathy (SCM).^[51,52]^ In this study, MAPKAPK2 expression was downregulated in the peripheral blood of CAD patients and could serve as another independent risk factor for CAD prediction. Importantly, the direction of MAPKAPK2’s regulatory effects on lipid metabolism may vary across different tissues and disease contexts.

AGPAT3, a member of the AGPAT family (1-acylglycerol-3-phosphate-*O*-acyl-transferase), functions as a lysophospholipid acyltransferase and is a key enzyme in the biosynthesis of phospholipids and triglycerides.^[53]^ Studies have found that phospholipids and triglycerides play important roles in tumorigenesis and development.^[54]^ Scp2 (sterol carrier protein 2) is a nonspecific lipid transfer protein crucial for the intracellular transport and metabolism of phospholipids, fatty acids, and cholesterol,^[55]^ and regulates angiogenesis, cell proliferation, and tumor migration.^[56]^ SCP2 facilitates inter-membrane lipid transfer by directly interacting with membrane lipids and promoting their desorption.^[57]^ As an apolipoprotein that can mediate cholesterol transport, SCP2 plays an important role in the development of hyperlipidemia, which is a major risk factor for atherosclerosis. SCP2 deficiency in LDLR^−/−^ mice significantly elevated high-densitylipoprotein cholesterol level while reducing very low-density lipoprotein, LDL-C, and triglyceride levels.^[58]^ Cholesterol accumulation in macrophages drives foam cell formation, a process in which SCP2 acts as a negative regulator of cholesterol efflux and plays a key role.^[59,60]^ Supporting this, SCP2 overexpression markedly increased hepatic cholesterol content in mice,^[59]^ while SCP2 knockout reduced plasma cholesterol and triglyceride levels and attenuates atherosclerotic plaque formation in LDLR-deficient mice.^[60]^ In this study, AGPAT3 and SCP2 were coordinately downregulated in the peripheral blood of CAD patients, suggesting that impaired phospholipid remodeling may significantly contribute to CAD pathogenesis.

TNFAIP8 (tumor necrosis factor-α induced protein 8) is a pro-tumorigenic factor with antiapoptotic biological function. It promotes cell proliferation and invasion through inhibiting apoptosis, thereby playing an important role in the occurrence and development of mutiple tumor types, including hepatocellular carcinoma, lung cancer, breast carcinoma, colorectal cancer, prostatic cancer, and etc.^[61-64]^ Furthermore, TNFAIP8 can target ATG3 (autophagy related gene 3) to regulate cellular autophagy function and simultaneously promote the secretion of inflammatory cytokines such as IL-6, IL-1β, and TNF-α, thus playing a significant role in immune responses and inflammatory reactions.^[65]^ We observed significantly reduced TNFAIP8 expression levels in the peripheral blood of CAD patients, consistent with trends observed in some autoimmune diseases. For instance, Filkor et al discovered the expression of TNFAIP8 was significantly downregulated in dendritic cells within the epidermal and dermal tissues of both lesional and non-lesional skin in psoriasis patients.^[66]^ Sun et al found that TNFAIP8-deficient mice exhibited more severe inflammatory clinical symptoms in mouse models of inflammatory colitis.^[67]^ One possible mechanism is that the failure of autophagy-dependent control over inflammatory cytokine production may contribute to the onset and progression of these diseases. Currently, research on TNFAIP8 in inflammatory and immune-related diseases is limited, and its specific mechanism in the development and progression of CAD requires further investigation.

Mechanistically, the identified biomarkers appear to converge on key biological processes implicated in CAD pathogenesis, including lipid transport, inflammatory signaling, and metabolic homeostasis. For example, HMGCR regulates cholesterol biosynthesis and is the pharmacological target of statins, whereas MAPKAPK2 participates in inflammatory signaling pathways linked to atherosclerotic plaque progression. SCP2 and AGPAT3 are involved in lipid trafficking and phospholipid remodeling, processes closely associated with lipid accumulation and vascular inflammation. TNFAIP8 has been implicated in immune regulation and inflammatory responses, suggesting potential involvement in chronic vascular inflammation underlying CAD development. Nevertheless, the precise molecular mechanisms require further experimental validation.

The mechanistic roles of both upregulated and downregulated genes in the onset and progression of CAD warrant careful consideration. Targeting highly expressed genes through the design of appropriate inhibitors represents a promising therapeutic strategy for CAD. Conversely, increasing the expression of downregulated genes via their agonist may help improve prognosis in CAD patients. Collectively, the reliably causal, lipid metabolism-associated marker genes identified in this study can predict CAD risk with high accuracy, indicating their potential as ideal therapeutic targets. Properly leveraging their value may hold promising application prospects for the future diagnosis and treatment of CAD. However, this study has several inevitable limitations. First, the DEGs data originated from a publicly available dataset specific to adult Filipinos, potentially limit the applicability of the results to other populations. Second, transcript levels may not fully reflect protein expression, therefore, continuous researches on the functional roles of these potential biomarker genes in regulating lipid metabolism during CAD should be conducted.

Despite the strengths of MR in inferring causality, several limitations should be acknowledged. First, horizontal pleiotropy may bias causal estimates if genetic instruments influence CAD through pathways independent of lipid metabolism. Although MR-Egger regression and sensitivity analyses suggested minimal pleiotropic effects, undetected pleiotropy cannot be completely excluded. Second, weak instrument bias may influence effect estimates despite stringent SNP selection criteria. Third, population stratification and residual confounding may still exist in GWAS datasets. Therefore, MR-derived causal relationships should be interpreted cautiously and ideally complemented by experimental validation. The transcriptomic dataset (GSE250283) analyzed in this study was derived from adult Filipino individuals, which may limit the generalizability of our findings to other ethnic populations. Ethnic differences in genetic architecture, environmental exposure, lifestyle factors, and cardiovascular risk profiles may influence gene expression patterns and CAD susceptibility. Although our MR analyses were based on large-scale GWAS datasets that improve causal inference robustness, further validation using multi-ethnic cohorts is required to confirm the universality of these biomarkers.

## 5. Conclusion

This study combined transcriptomic analysis with MR methods to systematically investigate the causal relationship between the eQTL data of 700 CAD-associated DEGs and CAD. After intersecting with the KEGG lipid metabolism gene set, 19 key genes exhibiting both lipid regulatory characteristics and reliable causal associations with CAD were obtained. Subsequently, 4 ML algorithms were employed to optimize biomarker selection, yielding a panel of 5 genes proven to predict CAD risk with high accuracy. The work presents novel and impactful findings that advance the understanding of CAD’s genetic underpinnings and lipid metabolism mechanisms. Furthermore, the innovative methodological framework of integrating transcriptomics, MR analysis, and ML methods provides powerful new tools for complex disease research. These breakthroughs herald a new era for cardiovascular disease prevention and treatment characterized by “mechanism-driven understanding → algorithmic optimization → precise implementation strategies,” particularly in identifying novel biomarkers for the early detection and therapeutic targeting of CAD.

## Author contributions

**Conceptualization:** Qi Bure, Wenjin Sun, Lujiao Wang, Xin Zhang, Lian Shuang.

**Data curation:** Qi Bure, Wenjin Sun, Lujiao Wang, Xin Zhang, Lian Shuang.

**Formal analysis:** Qi Bure, Wenjin Sun, Lujiao Wang, Xin Zhang, Lian Shuang.

**Funding acquisition:** Lian Shuang.

**Investigation:** Lian Shuang.

**Writing—original draft:** Qi Bure, Lian Shuang.

**Writing—review & editing:** Qi Bure, Lian Shuang.

## Supplementary Material


